# Restoration of spinal cord injury: From endogenous repairing process to cellular therapy

**DOI:** 10.3389/fncel.2022.1077441

**Published:** 2022-11-29

**Authors:** Yaqi Wu, Zhijian Tang, Jun Zhang, Yu Wang, Shengwen Liu

**Affiliations:** Department of Neurosurgery, Tongji Hospital, Tongji Medical College, Huazhong University of Science and Technology, Wuhan, China

**Keywords:** spinal cord injury, repair, cell transplantation, mechanism, stem cell

## Abstract

Spinal cord injury (SCI) disrupts neurological pathways and impacts sensory, motor, and autonomic nerve function. There is no effective treatment for SCI currently. Numerous endogenous cells, including astrocytes, macrophages/microglia, and oligodendrocyte, are involved in the histological healing process following SCI. By interfering with cells during the SCI repair process, some advancements in the therapy of SCI have been realized. Nevertheless, the endogenous cell types engaged in SCI repair and the current difficulties these cells confront in the therapy of SCI are poorly defined, and the mechanisms underlying them are little understood. In order to better understand SCI and create new therapeutic strategies and enhance the clinical translation of SCI repair, we have comprehensively listed the endogenous cells involved in SCI repair and summarized the six most common mechanisms involved in SCI repair, including limiting the inflammatory response, protecting the spared spinal cord, enhancing myelination, facilitating neovascularization, producing neurotrophic factors, and differentiating into neural/colloidal cell lines.

## Introduction

**FIGURE 1:**
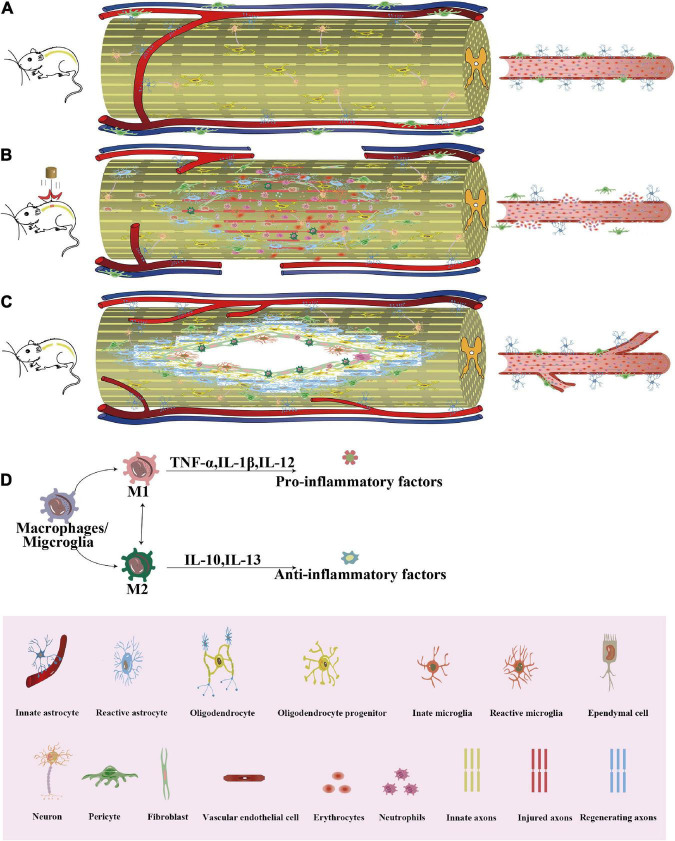
The process of cellular repair of SCI. After SCI, a series of repeated cascade responses, including cellular as well as non-cellular reactions, will occur. **(A)** Normal spinal cord and paraspinal vessels in uninjured mice. **(B)** In the acute phase of SCI, the BSCB is disrupted, neuronal death occurs, Wallerian degeneration of distal axons with a demyelinating response, permeability of the vessel wall increases, hematomas form, and hematogenous macrophages and neutrophils are recruited to the core of the lesion. Astrocytes migrate toward the core of the lesion and envelop the damaged tissue, thus protecting the remaining tissue from further damage. Microglia are also activated and morphologically altered to engulf the necrotic tissue along with blood-derived macrophages. Oligodendrocyte precursor cells have a marked ability to proliferate and interact with reactive glial cells at the border of the injury. Fibroblasts induced a marked fibroblast response, with large numbers of fibroblasts depositing around the core of the lesion. Epithelial cells migrated from the central canal of the spinal cord to the site of injury and participated in repair as endogenous stem cells. Pericytes detach from the basement membrane of the vessel wall and migrate to the injury site to participate in glial scar formation and to close the center of the injury. **(C)** In the chronic phase of SCI, the core of the lesion is often not effectively treated, resulting in the formation of a cavity in the lesion area surrounded by a glial scar formed by aggregates of hypertrophic astrocytes, pericytes, fibroblasts, and NG2+ oligodendrocytes, with neovascularization and accompanied by a small amount of myelin and axon formation. **(D)** The inflammatory environment causes changes in the macrophage phenotype. SCI, Spinal cord injury; BSCB, Blood-spinal cord barrier; TNF, Tumor necrosis factor; IL, Interleukin; NG2, Neuron-glial antigen 2.

Spinal cord injury (SCI) leads to a great burden for the patients and the society (Garcia-Altes et al., 2012; Dorsett et al., 2017; James Spencer and Theadom, 2019; Collaborators et al., 2021). The global incidence of SCI is 10.5 cases per 100,000 people, with an estimated 0.8 million new cases recorded each year (Kumar et al., 2018). Traffic accidents and falls have been considered to be the two most dominant causes all throughout the world (McDonald and Sadowsky, 2002; Alizadeh et al., 2019; Du et al., 2021). The spinal cord underwent two continuous pathophysiological processes after injury before entering a chronic state (McDonald and Sadowsky, 2002; Ahuja et al., 2017; Figure 1). The first stage is the primary injury caused by an external force, damaging the neural pathways and surrounding blood vessels (Alizadeh et al., 2019; Defrin et al., 2022). Then, a cascade of reactions including inflammation, oxidative stress, excitatory toxicity, ischemia, and astrocytic hyperplasia are triggered by the overflowing blood gradients and cellular components (Tran et al., 2018). All these changes lead to fibroglial scarring and cavity formation, as well as a further decrease of neurons and interruption of the neural pathways, which result in allodynia, impaired locomotor and autonomic dysfunction in some populations (Berger et al., 2014; Brommer et al., 2021; Defrin et al., 2022).

The pathological changes after SCI also induce an overproduction of molecules as chondroitin sulfate proteoglycans (CSPGs), Nogo A, myelin-associated glycoprotein, and oligodendrocyte myelin glycoprotein, which are inhibitors for neural regeneration in extracellular matrix (Moeendarbary et al., 2017). In animal models, researchers found that neutralization by functionally blocking antibodies, genetic deletion of Nogo-A, or blockade of Nogo-A receptors induces substantial axonal regeneration, as well as enhanced neuronal plasticity and functional recovery after SCI or stroke (Gonzenbach et al., 2010; Schwab and Strittmatter, 2014; Wahl et al., 2014). Furthermore, due to the lack of growth-promoting factors within the lesion, self-repair of the spinal cord to rebuild the damaged neural circuit is very difficult. So far as we know, effective approaches for the treatment of SCI patients are very limited. However, various strategies have been explored to treat SCI on animal models, including the transplantation of cells and biomaterials (Assinck et al., 2017a; Dumont et al., 2019), administration of medications (Liu et al., 2019) and neurotrophic factors (Xu et al., 2014), locomotor training (Angeli et al., 2018), and electrical stimulation (Formento et al., 2018). A great achievement has been made in animal experiments and some of these approaches have been applied in clinical trials for human subjects (Liu et al., 2019; Bartlett et al., 2020). For instance, in their initial human study published in 2018, Kucher academics assessed the early efficacy of human anti-nogo-A antibodies given intravenously to patients with acute, total traumatic paraplegia and quadriplegia. They discovered that following SCI, human anti-nogo-A antibodies partially recovered motor function. Their discoveries set the stage for the clinical use of anti-nogo-A antibodies. But more extensive studies on the effectiveness of anti-nogo-A antibodies in enhancing neurological recovery following SCI are required (Kucher et al., 2018).

At present, various treatments such as stem cell transplantation, growth factor injection, and biomaterial transplantation promote the repair of spinal cord function by reducing inflammatory response, promoting myelination, reducing cavity area, and promoting axon growth (Lu et al., 2014; Maldonado-Lasuncion et al., 2021). However, the complexity and heterogeneity of the pathophysiology of SCI are the main reasons for the lack of understanding and failure of SCI treatment. It is very crucial to comprehend the repair mechanism of SCI in order to offer a superior repair strategy (O’Shea et al., 2017). In light of this, this review summarizes the role of endogenous cells in the repair of SCI such as astrocyte, macrophages/microglia, oligodendrocyte, pericyte, endogenous neural stem/progenitor cell, Schwann cells, and other cells. In addition, we also summarized the targets of intervention through exogenous treatment of SCI, such as limiting the inflammatory response, protecting the spared spinal cord, enhancing myelination, facilitating neovascularization, producing neurotrophic factors, and differentiating into neural/colloidal cell lines. This review provides a very comprehensive description of the process of endogenous cellular repair in SCI and the associated mechanisms, which provides a solid basis for research strategies for SCI repair.

## Cell types involved in spinal cord injury repair

**FIGURE 2:**
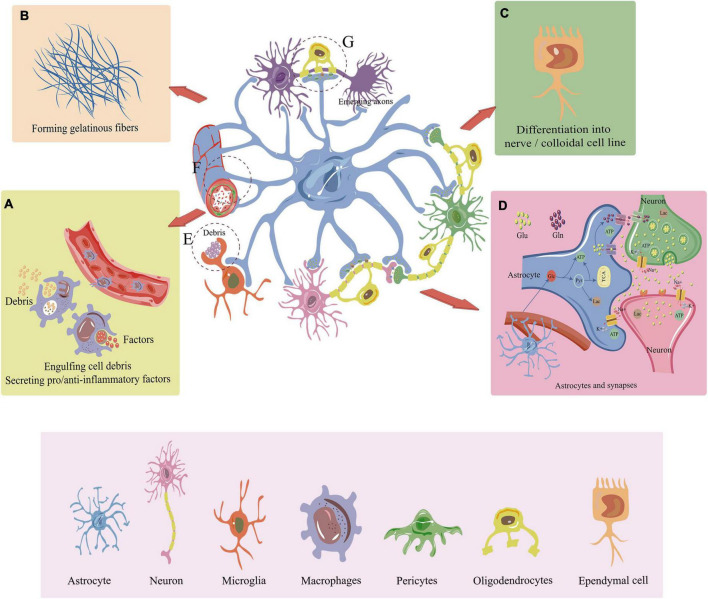
Major role of endogenous cells in the repair of SCI. **(A)** Under the influence of chemokines, macrophages migrate to the site of injury with outstretched peduncles to engulf cell debris and remnant tissues. They also stimulate relevant pro-inflammatory and anti-inflammatory molecules involved in the repair of spinal cord injuries. **(B)** Astrocytes and pericytes can secrete fibronectin to promote glial. **(C)** Ependymal cells are the major endogenous stem cells that differentiate into neural/colloidal cell lines that includes neurons, oligodendrocytes, and astrocytes. **(D)** Astrocytes take up glucose from blood vessels and convert it to lactate and ATP to provide energy for peripheral neurons. Astrocytes also facilitate the transmission of neurotransmitters such as glutamate, enhancing the signal communication between neurons. In addition, astrocytes maintain ion homeostasis inside and outside the cell. **(E)** Microglia engulf cellular debris and residual tissue at the site of SCI. **(F)** Astrocytes control blood flow by interacting with blood arteries. Pericyte can promote vascular remodeling, maintain normal vascular structure and stabilize the BSCB during SCI repair. **(G)** Oligodendrocytes help myelin regenerate, and astrocytes also give myelin and axon regeneration energy. SCI, Spinal cord injury; BSCB, Blood-spinal cord barrier; Glu, Glucose; Glu, Glutamate; Gln, Glutamine; TAC, Tricarboxylic acid cycle; Lac, Lactate; Pyr, Pyruvic acid.

A variety of cell types attempt to aid in the healing of SCI, and these cells interact with one another and take part in the healing process (O’Shea et al., 2017). We can create more efficient treatment plans by better understanding how cells repair it. These cells include astrocytes, macrophages/microglia, oligodendrocytes, pericytes, endogenous neural stem/progenitor cells, Schwann cells, and other cells, as described in Table 1 and Figure 2. The role that each type of cell involved in the repair process of SCI is described as follows.

**TABLE 1:** Cells involved in the repair process of SCI and their repair roles.

Types of cells	Study	Journal	Restoration effect
Astrocytes	Noristani et al., 2016	Molecular Neurodegeneration	Forming neurons and oligodendrocytes
	Wang et al., 2021	Cell	
	Xia et al., 2020	Translational Neurodegeneration	
Astrocytes	Bazargani and Attwell, 2016	Nature Neuroscience	Regulating blood flow
	MacVicar and Newman, 2015	Cold Spring Harbor Perspectives in Biology	
Astrocytes	Magistretti and Allaman, 2018	Nature Reviews Neuroscience	Providing energy to the surrounding neurons
Astrocytes	Klapper et al., 2019	Glia	Involving in the formation of synapses
Astrocytes	Parpura et al., 2016	Glia	Maintaining extracellular ion balance and delivering of related neurotransmitters
Astrocytes	Sofroniew and Vinters, 2010	Acta Neuropathologica	Creating glial scars at the site of injury
Astrocytes	Boddum et al., 2016	Nature Communications	Supporting axonal regeneration by regulating the release of synaptic active molecules
	Gundersen et al., 2015	Physiological Reviews	
Astrocytes	Torper et al., 2013	Proceedings of the National Academy of Sciences of the United States of America	Possessing specific stem cell characteristics
Macrophages/microglia	Liddelow et al., 2017	Nature	Secreting pro-inflammatory factors
Macrophages/microglia	David and Kroner, 2011	Nature Reviews Neuroscience	Secreting anti-inflammatory factors
	Hu et al., 2015	Nature Reviews Neurology	
	Orihuela et al., 2016	British Journal of Pharmacology	
	Zhou et al., 2014	Neural Regeneration Research	
Macrophages/microglia	Greenhalgh and David, 2014	Journal of Neuroscience	Phagocyticing and removing accumulated cellular debris and leftover tissue after SCI
	Greenhalgh et al., 2018	PLoS Biology	
Macrophages/microglia	Ikezumi et al., 2011	Histopathology	Promoting tissue fibrosis
	Pechkovsky et al., 2010	Clinical Immunology	
Oligodendrocytes/ oligodendrocyte progenitor cells	Kirby et al., 2019	Nature Communications	Promoting axon regeneration
Oligodendrocytes/ oligodendrocyte progenitor cells	Gibson et al., 2014	Science	Promoting myelin formation
Pericytes	Attwell et al., 2016	Journal of Cerebral Blood Flow and Metabolism	Constricting blood vessels
Pericytes	Fawcett et al., 2011	Nature Medicine	Forming scar tissue
	Goritz et al., 2011	Science	
Pericytes	Dias et al., 2018	Cell	Promoting fibrosis formation and extracellular matrix deposition
Pericytes	Chen et al., 2017a	Proceedings of the National Academy of Sciences of the United States of America	Maintaining the stability of the blood-brain barrier
	Yao et al., 2014	Nature Communications	
Pericytes	Hesp et al., 2018	Journal of Neuroscience	Promoting vascular remodeling
	You et al., 2014	Angiogenesis	Maintaining normal vascular structure Promoting tissue healing
Pericytes	An et al., 2022	Diabetes	Maintaining normal vascular structure
	Santos et al., 2019	Neuroscience Bulletin	
Pericytes	Rustenhoven et al., 2017	Trends in Pharmacological Sciences	Phagocytosis
Pericytes	Cheng et al., 2018	Acta Neuropathologica	Secreting neurotrophic factors
	Nikolakopoulou et al., 2019	Nature Neuroscience	
Pericytes	Nakagomi et al., 2015	Stem Cells	Possessing specific stem cell characteristics
Endogenous neural stem/progenitor cells	McKay, 1997	Science	Self-renewing and differentiating into neurons, astrocytes, and oligodendrocytes
	Reynolds and Weiss, 1992	Science	
	Stenudd et al., 2015	JAMA Neurology	
Schwann cells	Boerboom et al., 2016	Frontiers in Molecular Neuroscience	Promoting myelin formation
	Jessen et al., 2015	Cold Spring Harbor Perspectives in Biology	
	Norrmen et al., 2018	Journal of Neuroscience	
	Soto and Monje, 2017	Glia	
Schwann cells	Min et al., 2021	Glia	Recruiting macrophages
	Yin et al., 2022	Molecular Medicine	
Schwann cells	Jessen and Mirsky, 2016	The Journal of Physiology	Removing Waller degeneration and distal neuronal myelin and stimulating axonal growth
Schwann cells	Catignas et al., 2021	Glia	Secreting neurotrophic factors
	Maugeri et al., 2020	International Journal of Molecular Sciences	
	Selvaraj et al., 2012	Journal of Cell Biology	
	Sukhanov et al., 2021	Journal of Neuroscience	
Schwann cells	Liu et al., 2017a	Acta Biomaterialia	Promoting axon regeneration
Fibroblasts	Lee et al., 2020	Elife	Converting to inducible motor neurons
Microvascular endothelial cells	Zhou et al., 2019	Nature Neuroscience	Engulfing myelin fragments
Immunological T cells	Ishii et al., 2013	Cell Death and Disease	Initiating T cell-mediated autoimmune responses Secreting neurotrophic factors
Platelet cells	Ye et al., 2021	Neural Regeneration Research	Enhancing the performance of the BSCB

### Astrocyte

During the development of the mammalian nervous system, neurogenesis often occurs in the embryonic stage, while glial are formed after birth (Eze et al., 2021). Astrocytes are the complex and abundant glial cells in the nervous system, which are present throughout the spinal cord (Wiltbank and Kucenas, 2021). When cultured *in vitro*, activated astrocytes can form neurons, oligodendrocytes, and astrocytes under certain factors (Noristani et al., 2016; Xia et al., 2020; Wang et al., 2021). Astrocytes have a variety of functions, including the regulation of blood flow (MacVicar and Newman, 2015; Bazargani and Attwell, 2016), the provision of energy to surrounding neurons (Magistretti and Allaman, 2018), the formation and function of synapses (Klapper et al., 2019), maintenance of extracellular ion balance and the delivery of related neurotransmitters (Parpura et al., 2016).

The role of astrocytes in SCI is still controversial (Escartin et al., 2021). According to the majority of studies, SCI can activate astrocytes, which create glial scars at the site of injury that can wrap around injured tissue, reduce inflammation and prevent neuronal death, and maintain the integrity of the cells around the lesion (Robel et al., 2011). These reactive astrocytes are important for enveloping the injury site in the early stages of damage. Astrocytes can also play a direct role in synaptic transmission and may even support axonal regeneration by regulating the release of synaptic active molecules such as glutamate, purine (ATP and adenosine), GABA, and D-serine (Gundersen et al., 2015; Boddum et al., 2016; Figure 2). However, Yiu and other researchers believed that the persistent role of these glial scars would not only act as a physical barrier but also secrete neuroinhibitory factors that prevent the regeneration of axons, which is not conducive to the clinical repair of SCI (Silver and Miller, 2004; Yiu and He, 2006). Menet also demonstrated that reducing reactive astrocytes during injury improved axonal regeneration and enhanced functional recovery after SCI in mice lacking both GFAP and vimentin (Menet et al., 2003).

Astrocytes can be reprogrammed and a patient’s endogenous glial cells can be converted into neurons to treat SCI. Horner demonstrated that astrocytes lose their ability to form neurons after the second week of formation (Horner et al., 2000), but Vierbuchen et al. found that mature reactive astrocytes can still form neurons (Buffo et al., 2008; Vierbuchen et al., 2010; Caiazzo et al., 2011). Torper also demonstrated that astrocytes can be transformed into neuroblasts with proliferative properties by a single transcription factor SOX2 and further develop into functionally mature neurons *in vivo* (Torper et al., 2013). These findings imply that astrocytes might possess specific stem cell characteristics, opening up fresh avenues for the therapy of SCI. However, cell reprogramming is inefficient and the number of neurons successfully converted is relatively small currently. Due to the regional heterogeneity of astrocytes (Tsai et al., 2012), there are no specific studies of astrocytes in the adult spinal cord that can be reprogrammed *in vivo*. In order to enhance the reprogramming process and produce particular subtypes of neurons that support SCI repair, more study is required in this field, which necessitates the development of a novel, precise programming technique.

### Macrophages/microglia

Macrophages and microglia emerge from separate embryonic sources (Ginhoux et al., 2010; Schulz et al., 2012; Kierdorf et al., 2013). Mice’s microglia, which are resident macrophages in the central nervous system (CNS), are produced from erythromyeloid progenitors in the fetal yolk sac during embryonic development (Prinz and Priller, 2014; Bian et al., 2020). Macrophages are derived from extravasated monocytes, which are first made by aorta-gonad-mesonephros erythromyeloid progenitors (Perdiguero et al., 2015). Microglia and blood-derived macrophages are the sources of macrophages in the region of the lesion after spinal cord damage (David and Kroner, 2011).

Spinal cord injury induces a powerful and highly coordinated inflammatory response involving rapid activation and migration of microglia, accompanied by infiltration and recruitment of macrophages derived from peripheral blood monocyte within the lesion (David and Kroner, 2011). The damaged spinal cord tissue releases cytokines and chemokines into the circulation, and monocytes then migrate to the site of injury and differentiate into macrophages in response to the chemokines (Shechter et al., 2009). Inflammatory response after SCI is a double-edged sword (Plemel et al., 2014a; Gadani et al., 2015). Along with the death of neurons after SCI, the inflammatory response also causes glial scarring and cavitation. Recent research has however demonstrated that inflammation promotes neuronal regeneration and functional recovery, with activated microglia and blood-derived macrophages playing a crucial part (Cunha et al., 2020). Microglia and blood-derived macrophages primarily phagocytose and remove cellular debris and leftover tissue that accumulate after SCI (Figure 2). Microglia rapidly activate and proliferate after SCI, yet in the rat SCI model, blood-derived macrophages do not reach the SCI site until about 3 days after SCI and peak at 7 days after SCI, with two subsequent peaks at day 14 and day 60 after SCI, respectively (Chio et al., 2021). Therefore, compared to blood-derived macrophages, microglia have a much better efficiency and proliferation rate when phagocytosing lesioned myelin. When a mouse’s spinal cord was injured, macrophages were found toward the lesion’s center, whereas microglia were mostly found at the lesion’s perimeter (Shechter et al., 2009; Greenhalgh and David, 2014; Zrzavy et al., 2021; Figure 1). Additionally, it has recently been demonstrated that microglia and macrophages interact, and that co-culturing microglia and blood-derived macrophages causes a decrease in the capacity of the microglia to phagocytose myelin and an increase in the ability of the blood-derived macrophages to do so (Greenhalgh and David, 2014; Greenhalgh et al., 2018).

Microglia and blood-derived macrophages in SCI are mainly classified into two phenotypes according to their different cellular markers and functions: M1 type with pro-inflammatory properties and M2 type with anti-inflammatory properties (David and Kroner, 2011; Zhou et al., 2014; Hu et al., 2015; Orihuela et al., 2016; Figure 1). After SCI, M1 microglia and blood-derived macrophages upregulate and produce TNF-α, IL-1β, and chemokines, which draw inflammatory cells to the lesion to speed up the removal of necrotic tissue while simultaneously accelerating neuronal death and tissue damage (Liddelow et al., 2017). M2 microglia and blood-derived macrophages secrete TGF-β, IL-4, and IL-10, which suppress excessive immune inflammatory responses and promote the repair of damaged tissues (Cherry et al., 2014). Determining the conditions that will cause the phenotypic flip of M1/M2 macrophages is thus another issue that has to be addressed. One of the key strategies in the treatment of SCI will be to reduce the activation of M1-type cells while preserving the activation of M2-type cells or encouraging the conversion of M1-type to M2-type macrophages. M2 macrophages are important in tissue fibrosis as well (Pechkovsky et al., 2010; Ikezumi et al., 2011), therefore their prolonged presence after SCI may encourage the development of fibrotic scarring, which is harmful to axonal regeneration.

Due to their comparable shape and phenotypic characteristics in the diseased CNS, microglia and blood-derived macrophages are difficult to distinguish from one another. Transcriptomics revealed that specific genes such as TMEM119 and P2RY12 are highly expressed in microglia compared to blood-derived macrophages, so this can be used as a microglia-specific marker, but the levels of these markers are not stable and constant (Haynes et al., 2006; Bennett et al., 2016), so markers that can stably distinguish between macrophages and microglia need to be further explored by a wide range of scholars. Additionally, in patients who have had SCI, cellular debris may continue to exist for a number of years (Becerra et al., 1995). Therefore, a deeper comprehension of the processes by which microglia and blood-derived macrophages are ingested and digested is essential, and the phagocytosis of microglia and blood-derived monocytes may also be a viable target for the treatment of SCI.

### Oligodendrocyte

Myelin, the primary component of the myelin sheath, surrounds axons in the nervous system and is crucial for allowing axonal signaling (Figures 1, 2), which is the exchange of information between neurons (Stadelmann et al., 2019). The gray and white matter of the spinal cord includes oligodendrocyte progenitors that support the growth of endogenous oligodendrocytes and myelin regrowth (Maldonado et al., 2013; Gibson et al., 2014; Huang W. et al., 2020). By preventing inflammatory molecules from being released and secreting neurotrophic substances, oligodendrocyte progenitors aid in axonal regeneration (Kirby et al., 2019). The microenvironment following SCI, however, has an impact on its capacity to develop into oligodendrocytes (Alizadeh and Karimi-Abdolrezaee, 2016; Yalcin and Monje, 2021). Understanding the factors that promote endogenous oligodendrocyte progenitor cell activity in the SCI microenvironment can help to develop more effective repair strategies to achieve myelin regeneration after SCI (Gautier et al., 2015).

After SCI, oligodendrocyte death, a secondary demyelination reaction, causes axonal damage and the loss of sensorimotor function (Plemel et al., 2014b; Duncan et al., 2020; Floriddia et al., 2020). Axonal action potential transmission is dependent on sodium ion channels in the axonal membrane (Berret et al., 2016, 2017). Demyelination decreases the distribution of sodium ion channels in the axonal membrane, which may impede or slow action potential transmission and result in functional defects (Lubetzki et al., 2020). However, there isn’t any conclusive direct evidence that demyelination is what’s causing the conduction failure following SCI (Duncan et al., 2018). For instance, even when axons are preserved after SCI, there is decreased transmission in the spinal cord, which is clinically referred to as spinal shock (Ziu and Mesfin, 2022). The regeneration of myelin sheath in oligodendrocytes and the recovery of action potential conduction were also inconsistent in time. The partial potential conduction can be recovered within the first two weeks following SCI (Beaumont et al., 2006). But two weeks after SCI, oligodendrocytes began to regenerate their myelin (James et al., 2011).

It is currently unclear how exactly oligodendrocyte apoptosis occurs following SCI, while it may be related to ischemia or an inflammatory reaction at the damage site (Alizadeh and Karimi-Abdolrezaee, 2016). Calcium can accumulate toxically in oligodendrocytes as a result of excitatory toxicity brought on by glutamate or ATP (Voccoli et al., 2014; Lecca et al., 2016; Barron and Kim, 2019; Paez and Lyons, 2020). Oligodendrocyte death is also facilitated by inflammatory mediators generated by infiltrating neutrophils and microglia (Zirngibl et al., 2022). Therefore, preventing oligodendrocytes from dying or encouraging oligodendrocyte progenitor cells to differentiate following SCI can aid in the recovery of function (Manley et al., 2017; Sankavaram et al., 2019).

Whether mature oligodendrocytes have the ability to promote myelin regeneration has not been unanimously concluded in the scientific community. Some researchers have long believed that oligodendrocytes can neither migrate nor regenerate myelin sheath after SCI since oligodendrocytes are post-mitotic and differentiated (Crawford et al., 2016). Recent research, however, has revealed that mature oligodendrocytes do take a role in myelin repair following injury (Macchi et al., 2020). These variations in outcomes can be attributable to various experimental animal models, cell lines, and demyelination damage types. Regenerated myelin, however, is frequently thinner and less regular than healthy myelin (Duncan et al., 2017). The diameter of the axon and the thickness of the regenerated myelin sheath did not correlate linearly (Ludwin and Maitland, 1984). Additionally, myelin internodes are smaller and more delicate than usual (Griffiths and McCulloch, 1983). But Powers’ study contradicts it by arguing that past assessments have underestimated the extent and quality of regenerated myelin (Powers et al., 2013). Therefore, it is necessary to further confirm the difference between regenerated myelin and normal myelin at a later stage.

### Pericyte

**FIGURE 3:**
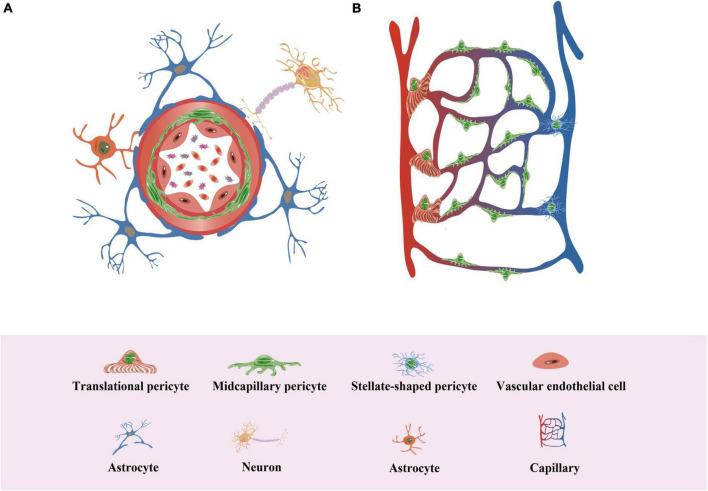
Pericytes in neovascularization. **(A)** Pericytes surround the endothelium in the vessel wall and are typically embedded in and linked to the basement membrane of the vessel. **(B)** Pericytes are a population of cells with various subtypes, including “Transitional pericyte”, which has more annular protrusions at the ends of small arteries in the capillary bed, “Midcapillary pericyte”, which has more longitudinal protrusions, and “Stellate-shaped pericyte”, which has more stellate protrusions at the ends of small veins in the capillary bed.

Pericytes are also called Rouget cells or parietal cells. Rouget made the initial discovery of pericytes in 1873 while researching the capillaries’ ability to contract (Rouget, 1873). In 1923, Zimmermann named them pericytes based on their location around blood vessels (Zimmermann, 1923). Pericytes, which are often embedded and linked to the blood vessel basement membrane, are found on the walls of blood arteries and surround endothelial cells (Attwell et al., 2016). Pericytes have different morphologies at different locations in the vasculature, including “Transitional pericyte,” which has more annular protrusions at the ends of small arteries in the capillary bed, “Midcapillary pericyte,” which has more longitudinal protrusions, and “Stellate-shaped pericyte,” which has more stellate protrusions at the ends of small veins in the capillary bed (Figure 3; Hartmann et al., 2015).

Astrocytes promoting scar formation after SCI have been a research hotspot (Anderson et al., 2016; Hara et al., 2017). Goritz and others discovered, however, that after SCI, pericytes detach from the basement membrane, multiply, and go to the SCI site where they take part in the creation of glial scar and seal the damage center (Figure 1; Fawcett et al., 2011; Goritz et al., 2011). Within 14 days of SCI in mice, the proliferation of pericytes exceeded that of astrocytes, and inhibition of pericyte proliferation disrupted scar formation and led to open tissue defects, which suggests that pericytes are also key to scar formation after SCI (Goritz et al., 2011). Additionally, it has been demonstrated that preventing pericyte proliferation lessens the fibrotic response and extracellular matrix deposition, which boosts axonal regeneration and encourages sensorimotor recovery after SCI (Dias et al., 2018). These studies have shown the significance of pericytes in the development of scars following SCI. The mechanism of peripheral cell growth and migration from the vascular wall to the scar site following SCI, however, is poorly understood (Dias et al., 2021).

Pericytes come in a variety of subtypes, and each subtype performs a unique function during SCI (Zhu et al., 2022). CD146 pericytes maintain the stability of the blood-brain barrier (Chen et al., 2017a) and secrete cell-adhesion molecules that allow pericytes to attach to endothelial cells (Iacobaeus et al., 2017); NG2 pericytes can not only promote vascular remodeling, maintain normal vascular structure, but also promote tissue healing (You et al., 2014; Hesp et al., 2018); PDGFR-β pericytes are the source of scar formation after SCI and have the potential to block lesions (Dias et al., 2021). However, conducting specialized study on pericytes is challenging since there is no one marker that can identify the entire pericyte population.

Pericytes play a very intricate role in SCI. It not only affects the integrity of the blood-brain barrier by influencing the role of AQP4 at the end of foot processes of astrocytes (Gundersen et al., 2014; Yao et al., 2014) but also plays a key role in angiogenesis (Caporali et al., 2017; Teichert et al., 2017). At the same time, pericytes play a crucial part in the stability and structural maintenance of blood vessels; otherwise, the blood vessels have a different shape and are more likely to burst as microaneurysms (Santos et al., 2019; An et al., 2022). Pericytes phagocytosis has been shown by Rustenhoven to be effective in clearing harmful materials from the microcirculation (Rustenhoven et al., 2017). Neurotrophic factors including NGF, BDNF, and NT-3 are produced by pericytes, and these substances can help neurons develop (Cheng et al., 2018; Nikolakopoulou et al., 2019). Pericytes also possess stem cell properties and can differentiate into neurons, astrocytes, and oligodendrocytes (Nakagomi et al., 2015). Research continues into the mediators and process governing pericyte directional differentiation. After SCI, severe demyelination frequently takes place, which impairs the transmission of motor signals (Orr and Gensel, 2018). Fuente discovered that pericytes would alter the CNS’s myelin regeneration process and that abnormalities in pericytes would cause a delay in myelin production through the research of a mouse model missing pericytes (De La Fuente et al., 2017). These results suggest that pericytes may be the key cells involved in the SCI repair process.

Pericytes are therefore possible therapeutic targets for SCI. The therapeutic use of pericytes is somewhat constrained since there is no reliable way to recognize and separate the many subtypes of pericytes. In order to implement appropriate targeted therapeutics, researchers can expand pertinent studies in this field and investigate the precise functions played by various pericyte subtypes throughout the development of SCI.

### Endogenous neural stem/progenitor cells

**FIGURE 4:**
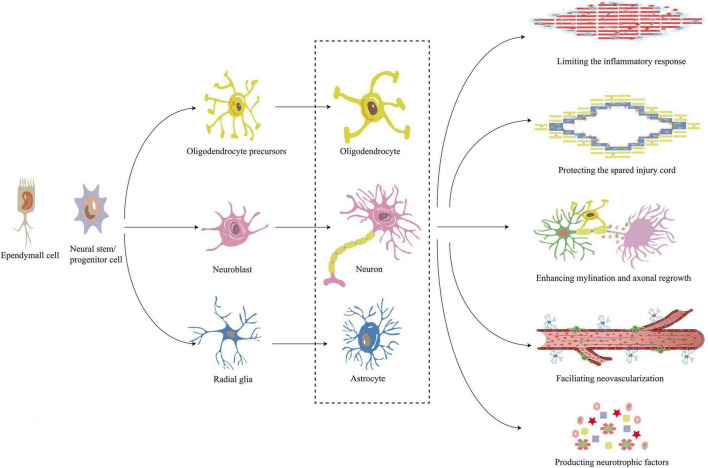
Mechanism of neural stem/progenitor cell and ependymal cell in repair of SCI. Neural stem/progenitor cell and ependymal cell differentiate into neural/colloidal cell lines including oligodendrocytes, neurons, and astrocytes. These differentiated cells work together to limit the inflammatory response, protect the spared spinal cord, encourage myelination, promote neovascularization and produce neurotrophic factors to promote the repair of SCI. SCI, Spinal cord injury.

In the adult spinal cords of rodents and primates, a region known as the neurogenic region has recently been identified. This region is made up of cells that surround the central canal, proliferate, and generate a range of cell types *in vivo* while acting similarly to neural stem cells *in vitro* (Weiss et al., 1996; Johansson et al., 1999; Horner et al., 2000; Meletis et al., 2008; Bamabé-Heider et al., 2010). Endogenous neural stem/progenitor cells are present in the nervous system with the ability not only to self-renew but also to differentiate into neurons, astrocytes, and oligodendrocytes (Figure 4; Reynolds and Weiss, 1992; McKay, 1997). Cells with endogenous neural stem/progenitor cells potential in the adult spinal cord, which lie dormant in the uninjured spinal cord, are activated and migrate to the injury center once the spinal cord is injured (Morest and Silver, 2003; Horky et al., 2006; Duncan et al., 2020). Activated neural stem cells (NSCs) can self-renew and differentiate into astrocytes and oligodendrocytes for tissue repair (Stenudd et al., 2015). Meletis has identified endogenous NSCs as ependymal cells through genetic profiling (Meletis et al., 2008). Ependymal cells are present in the ventricular system and the central canal of the spinal cord (Sabelstrom et al., 2014). In the mouse spinal cord, ependymal cells originate in the middle embryo and are already present completely around the central canal at birth (Li X. et al., 2016).

There are three main types of cells involved in uninjured spinal cord division, of which 80% were derived from NG2+/Olig2+ oligodendrocytes, <5% from GFAP+/CX30+/Sox9+ astrocytes and <5% from FoxJ1+ ependymal cells (Bamabé-Heider et al., 2010). Meletis divides ependymal cells into three basic types according to the form of them by Foxj1-Creer transgenic mice: Cuboidal ependymal cell, tanycyte, and radial ependymal cell (Meletis et al., 2008).

In animals with SCI, endogenous neurogenesis has almost completely repaired the damaged spinal cord, showing strong repair potential (Llorens-Bobadilla et al., 2020). After SCI, ependymal cells can proliferate, differentiate and migrate due to the change of microenvironment, such as the increase of some soluble factors, hypoxia, immune response, and so on (Perez Estrada et al., 2014; Covacu and Brundin, 2017). Activated ependymal cells mainly differentiate into astrocytes to form glial scar tissue that protects the integrity of remaining tissue and provides nutritional support for surviving neurons, making endogenous NSCs a potential therapeutic target in SCI. However, the ability of ependymal cells to differentiate into neurons is limited and the direction of differentiation is not controllable. At the same time, several studies claim that within the second decade of life, the whole cord almost completely lacks a patent central canal (Kasantikul et al., 1979; Milhorat et al., 1994; Yasui et al., 1999). In 2015, Garcia-Ovejero found by MRI, histology and immunohistochemistry, and laser capture microdissection that the central canal is predominantly absent in the adult human spinal cord, replaced by a structure that is morphologically and molecularly different from those described in rodents and other primates (Garcia-Ovejero et al., 2015). Therefore, the use of stem cell differentiation of ependymal cells for repair of SCI may be useful in immature adults, but not in adults. Additionally, the translation of therapeutic strategies for repairing SCI by ependymal cells in animal models to clinical studies should be approached with caution.

### Schwann cells

The neural crest is the origin of spinal nerve Schwann cells (Shi et al., 2016). Schwann cells are glial cells in the peripheral nervous system, which are distributed along with the processes of neurons and wrapped in nerve fibers to form myelinated nerve fibers (Nave and Werner, 2014; Salzer, 2015).

Because Schwann cells have the ability to dedifferentiate and re-differentiate and then re-myelinate when the nerve is damaged (Jessen et al., 2015; Boerboom et al., 2016; Soto and Monje, 2017; Norrmen et al., 2018). Therefore, transplantation of Schwann cells has been widely used in spinal cord demyelination models of rodents and primates to repair SCI and re-form myelin sheath to promote functional repair (Plemel et al., 2014b; Assinck et al., 2017a). Severe neurological problems can result from myelination disorders such demyelination, delayed myelination, or poor myelination (Duncan and Radcliff, 2016; Stadelmann et al., 2019). Despite these significant developments, transplanting Schwann cells only partially enhanced myelin regeneration (Assinck et al., 2017a; Monje et al., 2021), for which there are still no obvious explanations. Inadequate stimulation or inhibitory signals on re-differentiated Schwann cells may be the cause of hypomyelination. Furthermore, Sherman demonstrated that Schwann cells may have lost their normal response to myelin inducible factor (Sherman and Brophy, 2005). During the development of the peripheral nervous system, it is the axonal neuregulin-1 III that almost adjusted to all stages of the stem cell line, and the number of axial expressions of NRG1 III is determined to determine myeloid thickness (Bartus et al., 2016; Clark et al., 2017). Ruth showed that overexpression of axonal neuregulin-1 (NRG1) type III and NRG1 type I can restore normal myelination in transgenic mouse models of SCI (Stassart et al., 2013).

In addition to having a poor capacity for axon regeneration after SCI, the CNS also has a limited capacity for functional recovery. This might be the result of SCI-induced Waller’s degeneration of the distal nerve and demyelination of the nerve fibers, which causes an accumulation of numerous organelles and cell fragments at the lesion site and results in fluid-filled cavities and glial scars that prevent the regeneration of axons (Wozniak et al., 2018). Axonal regeneration is also hampered by the development of glial scars or matrix suppressors after SCI (Ghosh et al., 2012), in part because the buildup of CSPGs inhibits the migration of Schwann cells to the Waller degeneration region, which in turn affects immune cell response (Silver and Miller, 2004; Tran et al., 2018). Chondroitinase ABC can enhance the growth of axons in lesion sites and promote functional recovery by degrading the glycosaminoglycan (GAG) side chains of CSPGs (Kosuri et al., 2022). During Waller degeneration, chemokines and cytokines produced by Schwann cells can recruit macrophages, such as TNF-α, iNOS, and MCP-1 (Min et al., 2021; Yin et al., 2022), which help Schwann cells to clear Waller degeneration and distal neuronal myelination and stimulate axon growth to restore nerve conduction and function (Jessen and Mirsky, 2016).

Despite the fact that there are many other transplantable cells (Assinck et al., 2017a), Schwann cells are commonly regarded by researchers as the promising transplantation cell type for regeneration of spinal (Monje et al., 2021). Schwann cells have a long history of being transplanted, and the first transplantation experiment using pure Schwann cells was performed in 1981 (Duncan et al., 1981). The sensory function of the damaged spinal cord can be more effectively restored by Schwann cells (Poplawski et al., 2018; Rinwa et al., 2021). Schwann cells can secrete a variety of factors necessary to promote the survival of damaged neurons and axon regeneration, which can protect the residual tissue and promote the growth of axons. Such as ciliary neurotrophic factor (CNTF), cell adhesion molecules, pituitary adenylyl cyclase-activating peptide, and integrins (Selvaraj et al., 2012; Maugeri et al., 2020; Catignas et al., 2021; Sukhanov et al., 2021). Williams has demonstrated how transplanting Schwann cells may serve as a bridge and interact with astrocytes to encourage the outgrowth of axons in the lesion area (Williams et al., 2015). Our early research also demonstrated that the co-action of Schwann cells with regulated viral BDNF enhances axon regeneration in alginate hydrogels after SCI (Liu et al., 2017a). In 2017, Anderson scholars from the Miami Project to Cure Paralysis conducted a phase I clinical trial in which autologous human Schwann cells were transplanted into six patients with subacute SCI (Anderson et al., 2017). One year after transplantation, the patients had no surgical, medical, or neurological complications, and some patients with clinically significant neuropathic pain or muscular spasms were able to find some relief. In 2022, Gant again demonstrated the use of autologous Schwann cells for the treatment of chronic SCI, and they found that Schwann cells had significant efficacy in shrinking the diseased cystic cavity in patients with SCI (Gant et al., 2022). This confirms the great potential of Schwann cells for the treatment of SCI. Despite the fact that Schwann cells are found in the peripheral nervous system and not the CNS, following an SCI, these cells can migrate to the location of the damage through the spinal cord dorsal root (Zhang et al., 2013). The process by which Schwann cells enter the SCI region is unclear, nevertheless. People hypothesize that it might be caused by a variety of variables, including the development of blood vessels and an immunological response. In the early stages of Schwann cell transplantation for SCI, the functional recovery of SCI is not significant, and the therapeutic effect is not optimal (Hill et al., 2007), which may cause Schwann cells to die from necrosis and apoptosis due to damage to the microenvironment, low oxygen levels, M1-type macrophage-mediated inflammatory response, and cell-mediated immune response (Pearse et al., 2007). Existing evidence also suggests that axons rarely regenerate in the myelopathy area or in the area of cell transplantation during SCI. Even if the axon is regenerated, it cannot establish an effective connection with normal spinal cord tissue (Deng et al., 2013; Lee et al., 2016). This is one of the reasons why the region of SCI cannot be adequately improved by the implantation of Schwann cells. These studies also showed that Schwann cell transplantation alone was not enough to repair the regeneration of brainstem spinal cord axons. Therefore, researchers developed a number of elements that utilize combination treatment to boost the recovery of motor function (Anderson et al., 2018; Yang et al., 2021).

### Other cells

Fibroblasts, microvascular endothelial cells, neuron, and immunological T cells are additionally engaged in SCI repair in addition to the aforementioned cells (Table 1). By successively inducing POU5F1(OCT4) and LHX3, Lee demonstrated that human fibroblasts can be transformed into induced motor neurons (Lee et al., 2020). In mice with SCI, the transplantation of induced motor neurons into the injured area can aid in the recovery of motor function (Lee et al., 2020). Myelin sheath removal is very important to the function of nerve injury recovery, Zhou proved that IgG opsonization of myelin debris is required for microvascular endothelial cells engulfment myelin debris, myelin debris engulfment can induce endothelial-to-mesenchymal transition and the process to make the endothelial cells have the ability to stimulate the endothelial-derived production of fibrotic components (Zhou et al., 2019). The development of fibrotic scarring can help to reduce the inflammatory response to SCI and aid in the healing of spinal cord damage. Following SCI, CNS myelin-associated autoantigens (including myelin basic protein) can initiate T cell-mediated autoimmune responses, which can then produce a range of neurotrophic factors and cytokines in accordance with tissue requirements to aid in tissue repair (Ishii et al., 2013). Additionally, platelet cells might help the spinal cord heal from injury. Because Ye demonstrated that platelet-derived growth factor can enhance the blood-spinal cord barrier (BSCB)’s performance, boost the recovery of locomotor function following SCI, and promote endothelial cell regeneration by regulating autophagy (Ye et al., 2021).

## Cellular repair mechanisms of spinal cord injury

Schwann cells, NSCs, oligodendrocyte progenitors, olfactory sheathing cells, and mesenchymal stem cells (MSCs) are among the cell types that have been extensively researched for the therapy of SCI (Assinck et al., 2017a; Zipser et al., 2022). Ceto and his colleagues have also shown that transplanting neural or embryonic stem cells into damaged spinal cords can promote functional recovery (Assinck et al., 2017a; Ceto et al., 2020). Cell transplants were usually viable to treat SCI, according to clinical studies conducted in 2018 (Curtis et al., 2018). In this section, we outline six strategies by which cellular grafts repair SCI, including limiting the inflammatory response, protecting the spared spinal cord, enhancing myelination, facilitating neovascularization, producing neurotrophic factors, and differentiating into neural/colloidal cell lines (Figure 4). With a greater knowledge of these pathways, we may conduct pertinent research and potentially deliver matching targeted therapy, leading to better SCI treatment options.

### Limiting the inflammatory response

After SCI, the BSCB is breached (Jin et al., 2021), activating astrocytes (Karimi-Abdolrezaee and Billakanti, 2012), microglia/macrophages (Chen et al., 2017b), fibroblasts and other glial cells to encourage immune cell infiltration (Fernandez-Klett and Priller, 2014), as well as inducing complex inflammatory responses (Orr and Gensel, 2018; Hellenbrand et al., 2021), which leads to severe SCI.

Early inflammatory response, according to Cunha, is advantageous because it can assist eliminate tissue and cell debris and raise the amount of nutritional factors (Cunha et al., 2020). The large release of inflammatory factors, reactive oxygen species, proteolytic enzymes, and matrix metalloproteinases by inflammatory cells, however, can cause further harm to the surrounding normal spinal cord tissues when the inflammatory response persists (Neirinckx et al., 2014; Gadani et al., 2015). The fact that macrophages and microglia exhibit two opposing phenotypes, namely, neurotoxic M1 and anti-inflammatory M2, is proof of the complexity of the inflammatory response (Kigerl et al., 2009; David and Kroner, 2011; Miron et al., 2013). Kigerl reported that the number of M2-type macrophages would gradually decrease over time (Kigerl et al., 2009) and M1-type macrophages would play a dominant role after SCI, causing secondary SCI.

Drug administration, cell transplantation, and the use of biomaterials can change the microenvironment of SCI and create an anti-inflammatory state that can protect remaining tissue, promote repair, and enhance functional recovery (Gensel et al., 2017; Liu et al., 2017b). However, clinical measures to limit inflammation after SCI are very limited. The FDA has authorized the therapeutic medication methylprednisolone sodium succinate for SCI. However, due to its hazardous side effects, including gastrointestinal bleeding, aseptic necrosis of the femoral head, and wound infection, its clinical usage gradually decreased (Liu et al., 2019). Cell transplantation can promote the repair of spinal cord anatomy and function by alleviating adverse inflammatory responses. MSCs have been extensively researched in this area recently (Cofano et al., 2019; Andrzejewska et al., 2021). Professor Li implanted MSCs into the NT-3 gel sponge scaffold he invented and loaded it with neurotrophic factors, which enhanced the survival rate and anti-inflammatory effect of transplanted cells and thus promoted the regeneration of the spinal cord (Li G. et al., 2016). Exosomes from MSCs have the function of regulating the microenvironment. Li implanted exosomes secreted by MSCs into self-made hydrogels, making exosomes continuously released and effectively reducing inflammatory and oxidative reactions (Li et al., 2020). The use of biomaterials to control the diseased microenvironment is a potential approach to treating SCI (Lv et al., 2021; Silva et al., 2021).

Mesenchymal stem cells are multipotent progenitor cells with significant anti-apoptotic capabilities as well as the capacity to release a variety of neurotrophic and anti-inflammatory compounds. In this way, inflammation at SCI can be reduced and functional recovery can be promoted (Liau et al., 2020). When rats’ spinal injuries were treated with bone marrow MSCs, Nakajima’s team observed a switch from M1- to M2-type macrophages (Nakajima et al., 2012). The number of M1-type macrophages dramatically dropped at the same time that the number of M2-type macrophages significantly rose. In addition, the secretion of TNF-α and IFN-γ decreased while the secretion of IL-4 and IL-10 increased, which contributed to the reduction of cytotoxicity in the pathological microenvironment (Nakajima et al., 2012). Limiting inflammation may primarily be seen as a result of changes in macrophage phenotype following mesenchymal stem cell implantation (Bernardo and Fibbe, 2013). Future study will focus on determining the precise mechanism of action between them in order to determine if the limitation of the inflammatory response following SCI is caused by the transplanted cells, immunological control, or a combination of the two.

### Protecting the spared spinal cord

Primary SCI can cause complex secondary injury (O’Shea et al., 2017; Tran et al., 2018). Through many ways, cell transplantation may promote neuroprotective (Assinck et al., 2017a). The increase in normal tissue volume, the decrease in diseased tissue volume, and the release of neurotrophic factors are typical parameters used to assess the presence of neuroprotective effects. Numerous cell transplantation types, including Schwann cells, MSCs, and olfactory sheath cells, have been shown to have neuroprotective properties (Assinck et al., 2017a).

Cantinieaux et al. proved that transplantation of bone marrow MSCs in a rat model of SCI can not only reduce the size of spinal cord sac but also protect white matter tracts and reduce the inflammatory response, contributing to better motor function recovery (Cantinieaux et al., 2013; Kumagai et al., 2013; Mohammad-Gharibani et al., 2013; Wang et al., 2014). Transplantation of bone marrow MSCs after SCI can promote functional recovery to a certain extent, partly because of their neuroprotective ability to preserve neural tissues (Ritfeld et al., 2012). Transplantation of Schwann cells and olfactory sheath cells increased the number of spinal cord neurons and promoted the preservation of axons (Bunge, 2016; Gomez et al., 2018). Continuous spinal cord contraction occurs after SCI, resulting in increased volume of the damaged spinal cord (Ziegler et al., 2018). Lee believed that macrophage depletion and Schwann cell transplantation after SCI can also reduce cyst size (Lee et al., 2018). Laura’s team treated SCI with a combination of Schwann cells and gel significantly improved the spatial distribution of transplanted cells in the endogenous tissue. A reduction in cyst cavitation and neuronal loss was also observed, as well as a substantial increase in forelimb strength and coordination in mice (Marquardt et al., 2020).

Therefore, it is debatable whether lesion volume changes should be used to gauge the neuroprotective impact of transplanted cells. Many transplanted cells promote axonal regeneration and myelin sheath formation to increase the volume of remaining tissue (Powers et al., 2012; Hawryluk et al., 2014). Increased residual tissue is often associated with improved motor function (Basso et al., 1996; Schucht et al., 2002; Plemel et al., 2008). Therefore, the increase of residual tissue near the injury site can better explain the protective effect of cell transplantation on the injured spinal cord. At the same time, more researchers are needed to explore whether different stem cell types differ in their ability to increase the volume of tissue near the injury and protect the remaining tissue.

### Encouraging myelination

The tubular outer membrane that surrounds the axon of myelinated nerve fibers is referred to as the “myelin sheath” since myelin is its primary constituent. Insulation provided by myelin allows it to conduct impulse orientation by preventing interference between nerve impulses. In the event that an axon is destroyed, myelin can help direct the regeneration of the damaged axon. In the demyelinating disease, the nutritional support provided by myelin is essential for the survival of axons (Suminaite et al., 2019; Bolino, 2021).

In vertebrates, myelin loss and oligodendrocyte apoptosis (Crowe et al., 1997) that impair the conduction of nerve impulses (Funfschilling et al., 2012; Lee et al., 2012) can be seen in a few weeks after SCI. In an incomplete SCI, some degree of spontaneous re-myelination can be seen, which may be the result of myelination re-sheathed by transplanted Schwann cells or by oligodendrocyte precursors from other places (Duncan et al., 1981; Salzer, 2015; Assinck et al., 2017b). Duncan demonstrated that it is feasible to stimulate the development of endogenous progenitor cells into mature myelin-forming oligodendrocytes (Duncan et al., 2020).

Spinal cord injury in mammals has a limited ability to recover and to re-establish functional neural connections. Cell transplantation is currently a great way to repair myelin deficiency. After studying several biomaterials, we discovered that anisotropic alginate hydrogels can encourage axonal development and restore action potential conduction after SCI (Schackel et al., 2019; Huang L. et al., 2020). Oligodendrocytes are myelinated glial cells in the CNS, which can maintain the long-term integrity of axons (Simons and Nave, 2015). The majority of research have shown that the transplantation of NSCs or oligodendrocyte progenitor cells (OPCs) after SCI can improve myelin repair and functional recovery (Keirstead et al., 2005; Assinck et al., 2017a). These studies were unable to distinguish whether the restoration of function was due to neurotrophic support decreasing demyelinating harm or to transplanted cells remyelinating demyelinated axons. In 2005, Hofstetter’s research team transplanted adult NSCs into a rat model of thoracic SCI, which promoted the recovery of motor function and reduced myelin formation in the injured area, while leading to abnormal axonal growth and increasing the effect of ectopic pain (Hofstetter et al., 2005). This raises the possibility of substantial adverse effects from stem cell transplantation following SCI, raising doubts about the causal link between myelin regeneration and increased function. At the same time, most researchers believe that treating SCI with stem cell transplantation can alleviate neuropathic pain to some extent (Fandel et al., 2016; Hu et al., 2021). Since the experimental results are generally judged by the researchers based on the pain behavior of the mice, there is a lack of uniform standards for the mice’s response to pain, and the experimental results are also influenced by the subjective factors of the researchers and the heterogeneity of the mice.

Keirstead found that after transplantation of OPCs on day 7 or 10 months after SCI, only animals given OPCs on day 7 showed increased remyelination and improved motor function (Keirstead et al., 2005). This shows that the therapeutic window for remyelination is only available during the first few weeks following damage. This could also be because late astrocyte scarring prevents Schwann cell migration and axon regeneration, which affects re-myelination (O’Shea et al., 2017; Bradbury and Burnside, 2019). It’s intriguing that astrocytes can promote axon growth when they extend in a linear path to the site of the lesion (Matthews et al., 1979).

Most researchers studying spinal cord remyelination are unable to discriminate between intact and injured axons’ myelin state and their function. There are also three possible sources of remyelination after SCI: remyelination of endogenous NSCs (Powers et al., 2012; Hesp et al., 2015), myelination of transplanted cells (Sankavaram et al., 2019), and myelin preservation at the site of injury. The precise source of myelin regeneration following SCI is unclear at this time. Nashmi and Fehlings found in SCI models that most regenerated myelin sheaths were also incomplete, with new myelin sheaths being very thin and leading to conduction failure (Nashmi and Fehlings, 2001). Therefore, promoting myelin formation after SCI is not an accurate criterion for assessing functional recovery, unless there is an accurate method to determine the specific source of myelin in the tissue.

### Facilitating neovascularization

Spinal cord injury results in structural and functional changes of micro-vessels around the tissue (Li et al., 2017). Tao demonstrated that SCI was brought on by mechanical and physical action that cut off the spinal cord’s blood supply (Wang et al., 2018). The destruction of blood vessels not only reduces the perfusion of the remaining spinal parenchyma and destroys the BSCB, but also aggravates the ischemia, hypoxia, and inflammatory response of the injured spinal cord (McDonald and Sadowsky, 2002; Ahuja et al., 2017), which both hinder the repair of endogenous nerve tissue and aggravate SCI (Figley et al., 2014). Bearden and Rauch proved that the growth of blood vessels would guide the growth of axons (Bearden and Segal, 2004; Rauch et al., 2009). Lu’s research team also enlarged blood vessel diameter by atorvastatin, which increased blood perfusion and reduced neuron damage (Lu et al., 2004). Thus, increased blood flow appears to reduce neuron damage and provide better perfusion to damaged tissue (Yamaya et al., 2014). Vascular formation is also prior to functional recovery after SCI. Increased vascular density has been observed to be associated with improved recovery in many SCI models (Fassbender et al., 2011; Rocha et al., 2018; Hong et al., 2022). These studies indicated that the generation of spinal blood vessels may be a potential mechanism for spinal cord repair.

The expression of vascular endothelial growth factor (VEGF) can inhibit the apoptosis of vascular endothelial cells and promote the differentiation and migration of vascular endothelial cells (Sondell et al., 1999). It is a highly effective angiogenesis stimulator and vascular permeability regulator (Ferrara et al., 2003; Brockington et al., 2004). VEGF can not only directly protect neurons (Ku et al., 2017) and promote the growth of axons (Cattin et al., 2015), but also contribute to the survival and proliferation of a variety of glial cells (Sondell et al., 1999; Mani et al., 2005). In a mouse model of SCI, Deng observed that 3D human placenta-derived MSCs could produce trophic factors like VEGF and induce angiogenesis, indicating neuroprotective benefits (Deng et al., 2021). In addition, IL-8 can significantly increase the production of VEGF in BMSCs through PI3K/Akt and MAPK/ERK signaling pathways, which can enhance the angiogenic potential of BMSCs (Hou et al., 2014).

Mesenchymal stem cells have been used in clinical and preclinical trials for their role in promoting angiogenesis (Mathew et al., 2020). The treatment of human placental mesenchymal stem cell transplantation has also been proved by Kong to promote the repair of cerebral ischemia (Kong et al., 2018). Marrotte combined NSCs and endothelial cells with biomaterial hydrogel and successfully induced the generation of nerves and blood vessels in the rat SCI model (Marrotte et al., 2021). This biomaterial of biomimetic hydrogel has been proved by researchers to be able to induce the generation of blood vessels *in vivo* and *in vitro* (Sokic and Papavasiliou, 2012; Schweller et al., 2017). More and more studies have shown that stem cell transplantation for SCI is mainly mediated by exosomes secreted by stem cells (Mendt et al., 2019; Cao et al., 2021). Zhang extracted exosomes derived from human embryonic MSCs by centrifugation and injected them into the SCI model. They found that the number of blood vessels, vascular connectivity, and vascular volume fraction in the spinal cord significantly increased and improved motor and sensory function (Zhang et al., 2020).

The development and regeneration of blood vessels are associated to functional recovery after SCI, according to the results of the available study; however, the pertinent particular mechanism research is insufficient. Future study might go in this route because there is no pertinent literature to support the idea that the transplanted cell types affect vascular development differently.

### Producing neurotrophic factors

Neurotrophic factor and cytokines may play a part in the neurotrophic process of cell transplantation for SCI (Hawryluk et al., 2012a,b; Garcia et al., 2016). *In vitro*, trophic factors and cytokines can be secreted by MSCs, neural precursor cells (NPCs), Schwann cells, and microglia. After transplantation, these cells can also enhance the amount of these substances in the host (O’Shea et al., 2017; Maldonado-Lasuncion et al., 2018; Maugeri et al., 2020). Numerous healing effects are produced by neurotrophic factors and associated trophic factors. For example: apoptosis prevention (Zhang et al., 2014), axon regeneration promotion (Anderson et al., 2018), myelination enhancement (Bartus et al., 2016), and regulate of NPCs proliferation and differentiation (Gao et al., 2022). These healing effects are mostly interconnected. However, NGF can also have adverse effects, such as causing neuropathic pain (Dai et al., 2020).

The nutritional factors most commonly examined by researchers include NGF, BDNF, and NT-3 (Nieto-Sampedro et al., 1982; Dougherty et al., 2000; Widenfalk et al., 2001; Tokumine et al., 2003). After SCI, the expression of nutrient factors NGF, BDNF, GDNF, and so on are upregulated (Mocchetti and Wrathall, 1995; Dougherty et al., 2000; Widenfalk et al., 2001), while there are research works that show the opposite changes (Uchida et al., 2003; Gulino et al., 2004), which may be related to the SCI model, the severity of the damage, and the various nutritional factor detection times. Therefore, future research in this area may be better able to identify changes in nutritional factor expression following SCI using the same criteria.

Since cell transplantation has pleiotropic effects and neurotrophic advantages for the surrounding tissues, it is essentially a combined treatment for SCI healing. Hawryluk discovered that cell transplantation and the neurotrophic medications minocycline and cyclosporine worked best together to treat SCI (Hawryluk et al., 2012a). The use of neurotrophic factors incorporated into biomaterials to improve NSPCs transplantation for SCI has also shown impressive results (Li G. et al., 2016). However, there is little direct evidence that cells supporting transplantation produce nutritional factors. Different transplanted cells also produce different nutritional factors (Hawryluk et al., 2012b). The mechanism by which transplanted cells produce nutritional factors is unclear, and there have been few systematic research works on the association between the kind of transplanted cells and the cell-derived nutrients required for SCI healing. Therefore, it would be important to further understand in detail whether the secretion of nutritional factors and cytokines by transplanted cells is necessary for SCI repair.

### Differentiating into nerve/colloidal cell line

Neurons, astrocytes, and oligodendrocytes can all develop from NSCs, which are pluripotent stem cells (McKay, 1997; Barnabe-Heider and Frisen, 2008; Assinck et al., 2017a). The differentiation of NSCs is regulated by both the environment and its own characteristics. *In vivo* and *in vitro* NSC modulation is mediated by Notch and Rho signaling pathways (Ben-Shushan et al., 2015; Hosseini et al., 2022). There are more differentiated astrocytes than neurons in endogenous NSCs when Notch1 and Hes1 genes are highly expressed. Notch can inhibit, delay, or induce differentiation and can promote cell division and apoptosis through multiple pathways. When the Notch pathway is activated, stem cells would be proliferated; however, when the Notch signal is suppressed, stem cells would differentiate (Kageyama and Ohtsuka, 1999; Lai, 2004; Bhat, 2014; Pinto-Teixeira and Desplan, 2014). Meletis proved that transfection of Ngn2 and OligO2 can promote the differentiation of NSCs into motor neurons and oligodendrocytes, respectively (Meletis et al., 2008). This suggests that the process and proportion of stem cell differentiation into neural functional cells can be precisely regulated by changing the relevant signals.

Research has recently shifted its attention to the variables influencing the proliferation and development of NSCs. There have been numerous research works on how to induce stem cells to develop into neurons. We combined NSCs with alginate hydrogel cross-linked by Ca2+ and found that approximately one-third (38.3%) of the NSCs survived after transplantation into acute T8 complete spinal cord transection sites in adult rats and differentiated into neurons (40.7%), astrocytes (26.6%), and oligodendrocytes (28.4%) at 8 weeks post-transplantation (Zhou et al., 2022). Johe and Massey found that the complex interaction between matrix, medium and cells could affect the proliferation and differentiation of NSCs (Johe et al., 1996; Tsai and McKay, 2000). Li, Hung and Wang also proved that some matrix can guide the extension of nerve cells to the target (Hung et al., 2006; Wang et al., 2006; Li et al., 2009). BDNF and GDNF are two major neurotrophic factors, which are essential for the proliferation and differentiation of NSCs (Deng et al., 2013; Wakeman et al., 2014; Chang et al., 2021). Platelets-derived growth factor, ciliary neurotrophic factor, and sonic hedgehog have been shown to play an important role in stem cell survival and differentiation into specific neural lineages (Lachyankar et al., 1997; Caldwell et al., 2001; Bambakidis et al., 2003; Kondo et al., 2005; Li et al., 2005). These results indicate that neurotrophic factors and stroma can control the proliferation and differentiation of NSCs to a certain extent (Lachyankar et al., 1997; Wang et al., 2006).

## Prospect

The repair of SCI is a complex pathophysiological process. In recent years, a lot of research has been conducted and a certain degree of success has been achieved through strategies such as stem cell transplantation, biomaterials, and exosomes to promote SCI repair. However, due to the heterogeneity of SCI and the complexity of the repair process, no ideal repair strategy has been found for the repair and regeneration of SCI.

With stem cell therapy for SCI, people should pay attention to the variations among study methodologies, the effectiveness and purity of stem cell differentiation, and the development of teratomas (Nori et al., 2015). Low differentiation and survival rates of stem cells are typical issues with stem cell transplantation for SCI (Gericota et al., 2014; Iwai et al., 2014; Lee et al., 2014; Tuszynski et al., 2014). NSCs transplanted into the injured spinal cord differentiated mainly into glial cells (Cao et al., 2001; Zhou et al., 2022). More research is needed to achieve targeted differentiation of NSCs. Therefore, these challenges further exacerbate the heterogeneity of patients with SCI and hinder clinical research on stem cell therapy for SCI, which also needs to be addressed in basic research. Treatment of SCI by biomaterials requires consideration of biodegradation rate, biocompatibility, and safety of the material to facilitate better clinical translation. In addition, exosome therapy faces many challenges. To date, ultracentrifugation remains the most commonly used method for exosome isolation and concentration, and although there are various methods for exosome extraction, the recovery rate and specificity of exosome isolation still need to be improved, and ultracentrifugation destroys the integrity of exosomes and has a high rate of protein contamination. In clinical applications, the development of accurate and effective standard methods for the identification, isolation, and quantification of exosomes is still an urgent issue to be addressed. Combination therapy might be superior to single therapeutic approaches. Combination therapies like “biomaterials + stem cells + growth factors,” “exosomes + stem cells + growth factors,” and “biomaterials + exosomes + stem cells” have been successful in animal models, but the majority of the current animal experiments have been done in the acute and subacute phases of SCI, and the treatment of the chronic phase of SCI needs more research.

This review summarizes the cell types involved in the repair process of SCI and the common repair mechanisms of SCI, which can be very helpful for the clinical translation of SCI treatment. This review can provide a better understanding of the pathophysiological processes involved in SCI repair and help them to develop more targeted strategies for SCI repair.

## Author contributions

YqW illustrated the figures and wrote the manuscript. All authors provided the input and approved the submitted version.
